# The Biology of Vasopressin

**DOI:** 10.3390/biomedicines9010089

**Published:** 2021-01-18

**Authors:** Samantha Sparapani, Cassandra Millet-Boureima, Joshua Oliver, Kathy Mu, Pegah Hadavi, Tamar Kalostian, Nazifa Ali, Carla Maria Avelar, Marion Bardies, Brenton Barrow, Minky Benedikt, Giuliana Biancardi, Raminder Bindra, Lisa Bui, Zakaria Chihab, Ashley Cossitt, Jeffrey Costa, Tina Daigneault, Jocelyn Dault, Isa Davidson, Jonathan Dias, Emie Dufour, Sabine El-Khoury, Nargess Farhangdoost, Anika Forget, Alexa Fox, Myriam Gebrael, Maria Concetta Gentile, Olivia Geraci, Ansley Gnanapragasam, Elias Gomah, Elie Haber, Claudia Hamel, Thivya Iyanker, Christina Kalantzis, Sara Kamali, Elsa Kassardjian, Hryssi Krissy Kontos, Thi Bich Uyen Le, Daniella LoScerbo, Yan Fang Low, Danielle Mac Rae, Flore Maurer, Sana Mazhar, Alice Nguyen, Kathy Nguyen-Duong, Chelsea Osborne-Laroche, Hwi Wun Park, Emilie Parolin, Kahlila Paul-Cole, Leah Sarah Peer, Margaux Philippon, Charles-Alexandre Plaisir, Jessica Porras Marroquin, Simran Prasad, Rewaparsad Ramsarun, Saad Razzaq, Samantha Rhainds, Damien Robin, Ryan Scartozzi, Davindra Singh, Sajad Soleimani Fard, Maxim Soroko, Nastaran Soroori Motlagh, Kiri Stern, Laila Toro, M. Wyatt Toure, Stephanie Tran-Huynh, Sarah Trépanier-Chicoine, Claudia Waddingham, Aaliyah Jasmine Weekes, Allison Wisniewski, Chiara Gamberi

**Affiliations:** Biology Department, Concordia University, Montreal, QC H4B 1R6, Canada; samantha.spa@hotmail.com (S.S.); cassandra.millet@mail.concordia.ca (C.M.-B.); irishstrat312@outlook.com (J.O.); kathy-mu@hotmail.com (K.M.); p.hadavi@yahoo.com (P.H.); tamarkalostian@gmail.com (T.K.); a_naz20@hotmail.com (N.A.); carla.maria.avelar@gmail.com (C.M.A.); bardies2@hotmail.com (M.B.); b.barrow@hotmail.com (B.B.); benediktminky@gmail.com (M.B.); giulianabiancardi@gmail.com (G.B.); raminderbindra3@gmail.com (R.B.); li_bui@live.concordia.ca (L.B.); zadoukii@hotmail.com (Z.C.); Akcossitt@gmail.com (A.C.); jeffrey_costa@hotmail.com (J.C.); tinad722@gmail.com (T.D.); jocelyndault@gmail.com (J.D.); davidson.isa@gmail.com (I.D.); jonathan.dias370@gmail.com (J.D.); emie.dufour@gmail.com (E.D.); sabinekhoury999@hotmail.com (S.E.-K.); nargess_farhang@yahoo.com (N.F.); anikamoky2@hotmail.com (A.F.); alexa.fox88@gmail.com (A.F.); myriam_ed2012@yahoo.com (M.G.); mari_gentile@hotmail.com (M.C.G.); oli_geraci@hotmail.com (O.G.); ansley.g@hotmail.com (A.G.); ej.17_socca@hotmail.com (E.G.); elie_818@hotmail.com (E.H.); claudiaxkhamel@hotmail.com (C.H.); thivyaiyanker@gmail.com (T.I.); ckalantzis@hotmail.com (C.K.); sarah_kamali@yahoo.com (S.K.); elsa.kassardjian@gmail.com (E.K.); kontosk@hotmail.com (H.K.K.); uyenbright3011@gmail.com (T.B.U.L.); daniella.loscerbo@gmail.com (D.L.); anastasialow@gmail.com (Y.F.L.); daniellembmacrae@gmail.com (D.M.R.); flore.maurer@gmail.com (F.M.); mazharsana1@yahoo.com (S.M.); alicee.ng@hotmail.com (A.N.); katnd@hotmail.com (K.N.-D.); c.osborne.laroche@gmail.com (C.O.-L.); hwiwun@hotmail.com (H.W.P.); emiliep25@hotmail.com (E.P.); kahlilapc@hotmail.ca (K.P.-C.); leahsarahpeer@hotmail.com (L.S.P.); margaux.philippon@hotmail.fr (M.P.); plaisircharly@gmail.com (C.-A.P.); porras.jessica92@gmail.com (J.P.M.); forsimran@hotmail.com (S.P.); bbbhushan@hotmail.com (R.R.); razzsaad97@gmail.com (S.R.); Samantharhainds@hotmail.com (S.R.); damien.robin35@gmail.com (D.R.); ryan.scartozzi@gmail.com (R.S.); davin_44@hotmail.com (D.S.); s.sfard@yahoo.ca (S.S.F.); maxim.soroko@hotmail.com (M.S.); nastaransoroori04@hotmail.com (N.S.M.); kiri.a.stern@gmail.com (K.S.); torolaila@gmail.com (L.T.); wyatt290.2@gmail.com (M.W.T.); stephaniiee_92@hotmail.com (S.T.-H.); sarahtrep-chi@hotmail.com (S.T.-C.); claudiawaddingham@gmail.com (C.W.); aaliyah.weekes@hotmail.com (A.J.W.); awisniewski365@gmail.com (A.W.)

**Keywords:** vasopressin, renal function, cardiac function, social behavior, sex differences, GPCRs

## Abstract

Vasopressins are evolutionarily conserved peptide hormones. Mammalian vasopressin functions systemically as an antidiuretic and regulator of blood and cardiac flow essential for adapting to terrestrial environments. Moreover, vasopressin acts centrally as a neurohormone involved in social and parental behavior and stress response. Vasopressin synthesis in several cell types, storage in intracellular vesicles, and release in response to physiological stimuli are highly regulated and mediated by three distinct G protein coupled receptors. Other receptors may bind or cross-bind vasopressin. Vasopressin is regulated spatially and temporally through transcriptional and post-transcriptional mechanisms, sex, tissue, and cell-specific receptor expression. Anomalies of vasopressin signaling have been observed in polycystic kidney disease, chronic heart failure, and neuropsychiatric conditions. Growing knowledge of the central biological roles of vasopressin has enabled pharmacological advances to treat these conditions by targeting defective systemic or central pathways utilizing specific agonists and antagonists.

## 1. Introduction

Foreword: This review is the result of a didactic project at Concordia University in Montreal employing a novel “write to learn” pedagogy [1] that we have used successfully before [2,3]. Senior undergraduate students enrolled in a Comparative Physiology course for third-year Biology majors were taught experientially how to research and study the scientific literature to write a collaborative analytical review on the biological activity of vasopressin. We apologize to those colleagues whose important research could not be cited because of space or pedagogic constraints.

In humans and rodents, arginine vasopressin (AVP) regulates several diverse physiological functions: Fluid balance, blood osmolarity, reproduction, complex behavior, memory, and learning. An antidiuretic hormone, vasopressin is a nonapeptide conserved from invertebrates to vertebrates that displays species-specific amino acid (aa) changes in positions 3 and 8 [4]. Vasopressin is thought to have enabled the survival of land-dwelling organisms by regulating tubular reabsorption in the kidneys to maintain internal water homeostasis and blood osmolarity [5]. Here, we review the biology of AVP through examination of its normal physiological roles in the kidneys and heart, its neurological and behavioral effects, how AVP dysfunction contributes to pathologies such as polycystic kidney disease (PKD), heart failure (HF), neuropsychiatric disorders, and the pharmacological manipulation of AVP-dependent pathways.

### 1.1. Early Discoveries

The antidiuretic function of AVP was first demonstrated in 1913 by F. Farini in Venice and, independently, by von den Velden in Düsseldorf, who injected extracts from the posterior lobe of the pituitary gland into anesthetized men to control excessive water loss due to *diabetes insipidus* (a rare condition unrelated to type 1 diabetes (reviewed in [6]) or pituitary damage [7,8,9]. At the time, bovine pituitary extract was known for its oxytocic (triggers uterine contractions) and pressor (raises blood pressure) properties, albeit the underlying mechanisms were unknown. In 1927, two active components were isolated and called respectively α- and β-hypophamines (i.e., amines driven from the hypophysis). Their chemical synthesis shortly followed, and the resultant products were assigned the trade names oxytocin (OT; α-hypophamine or Pitocin) and AVP (β-hypophamine or Pitressin) [10,11]. AVP receptors (R) Avpr1a (today’s V1aR), Avpr2 (V2R), and Avpr1b (V1bR) were subsequently cloned, as well as the single oxytocin receptor Oxtr (OTR) [12,13,14,15,16,17,18,19]. 

### 1.2. AVP Function 

AVP secretion from the posterior pituitary is triggered by changes in the electrolyte-water balance affecting intravascular blood volume and osmolality, that respectively activate baroreceptors [20] and osmoreceptors [21]. When serum sodium (Na^+^) levels rise above 145 mmol/L, the resulting hypernatremia activates the hypothalamic osmoreceptors within the *organum vasculosum lamina terminalis* (OVLT) and the subfornical organ that signal to the supraoptic (SON) and paraventricular nuclei (PVN) and induce AVP secretion by the posterior pituitary into the bloodstream [5,22]. Changes in osmolality of the thalamic magnocellular cells activate nonselective cation channels, increase the action potentials firing rate, and trigger AVP release from axon terminals ([23], reviewed in [24]). Heightened hematic AVP leads to water reabsorption by the kidneys to dilute Na^+^ in the organism [20,21,23]. Conversely, decreased osmolality promotes AVP retention and increased water excretion [25]. AVP interacts with the transmembrane receptors V1aR, V1bR, and V2R expressed by several cell types [25,26,27]. Patients suffering from congestive heart failure (CHF) display high basal levels of AVP, which increases vascular smooth muscle tone [28,29,30]. Changes in blood volume, especially when paired to a drop in blood pressure, e.g., caused by hemorrhage, activate baroceptors, and can also induce AVP secretion [5]. In the brain, AVP functions as a neuropeptide regulating social behavior [31,32,33,34]. 

### 1.3. AVP Gene Expression 

The *AVP* gene expression is tightly controlled. The *AVP* promoter contains a cyclic AMP (cAMP) response element (CRE) recognized by the phosphorylated CRE-binding protein in response to increased intracellular cAMP [35,36,37]. The AP1 and AP2 transcription factors promote *AVP* transcription, while the glucocorticoid receptor represses it [38]. Furthermore, the *AVP* mRNA is regulated post-transcriptionally through polyadenylation. In the hypothalamic cells of salt-deprived rats, the *AVP* mRNA poly(A) tail length was longer than in non-salt-deprived rats [39,40]. Long polyA tails are likely to increase translation of the cognate protein [41]. In mammalian neurons, the poly(A) binding protein bound to the *AVP* mRNA on the “dendritic localization sequence” increased mRNA stability and translation [39]. 

### 1.4. AVP Synthesis 

**Figure 1:**
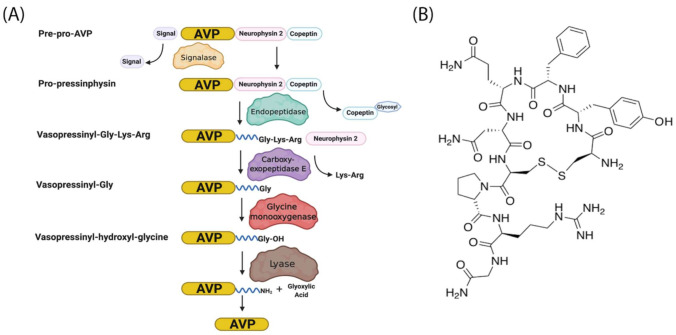
AVP (arginine vasopressin) synthesis and structure. (**A**) The hypothalamic neurosecretory neurons synthesize the pre-pro-hormone precursor called pre-pro-AVP or AVP-neurophysin-copeptin. The signal peptide is cleaved by a signalase in the endoplasmic reticulum to form pro-pressinphysin. Copeptin is glycosylated and cleaved by an endopeptidase in the Golgi [8,35,45]. Such endopeptidase also separates the pro-AVP vasopressinyl-Gly-Lys-Arg peptide from neurophysin, and pro-AVP is enclosed in vesicles. The C-terminal arginine and lysine are trimmed by carboxypeptidase E and the newly exposed C-terminal glycine is oxidized by glycine monooxygenase into hydroxyl-glycine. Finally, a lyase converts hydroxyl-glycine is into an amide group which subsequently reacts with glyoxylic acid to yield AVP [45]. (**B**) AVP structure. Note the cyclic structure and primary amide (NH_2_).

AVP is produced from a 164 aa long pre-pro-hormone precursor in the body of the magnocellular neurons of both PVN and supra-ventricular nucleus of the hypothalamus [42], and at lower levels in parvocellular neurons of the PVN [43]. Pre-pro-AVP contains an *N*-terminal signal peptide, followed by the AVP nonapeptide, and the open reading frames of regulatory peptides neurophysin-2 and copeptin [44]. Figure 1A illustrates the pre-pro-AVP processing and post-translational modification [38,45].

Pro-AVP is stored in membrane-associated granules and released in response to increased extracellular fluid osmolarity and osmoreceptor activation [25]. The threshold of AVP release and plasmatic AVP levels differ in men and women and among individuals [46,47]. Mature AVP is the cyclic nonapeptide Cys-Tyr-Phe-Gln-Asn-Cys-Pro-Arg-Gly-NH_2_ (reviewed in [42]) in which the two cysteines form a disulfide bridge, and the terminal carboxyl residue is modified post-translationally into a primary amide ([37,48], Figure 1B). AVP circulates as a free hormone and is degraded enzymatically in the liver and kidney within 10–30 min of release [49].

### 1.5. AVP Receptors 

V1aR, V1bR, and V2R are G protein-coupled receptors (GPCRs) that interact with trimeric G proteins, G_q/11_ and G_s_ [50]. Each receptor has distinct tissue expression. The BGee gene expression database [51] lists 168 human tissues expressing V1aR mRNA, 59 for V1bR, and 129 for V2R and species-specific relative variability. V1aR expression in the Suprachiasmatic nucleus (SCN) exhibits diurnal rhythmicity [52]. Functional information for organs and tissues relevant to this review is listed in Table 1. 

#### 1.5.1. Kidney

**Figure 2:**
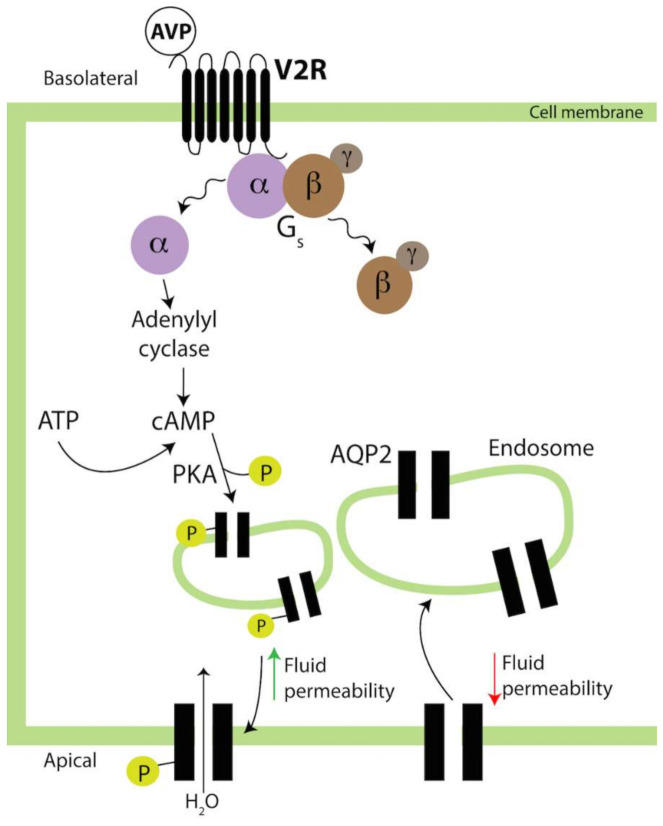
Intracellular response to AVP in renal tubular epithelial cells. AVP binds to the V2R G-protein coupled receptor on the basolateral membrane, which activates adenylyl-cyclase (AC). Produced cAMP activates protein kinase A (PKA), which phosphorylates the AQP2 water channels stored in vesicular compartments. This promotes apical membrane relocation and increased transmembrane water transport (modified after [95]).

The V1aR receptor is expressed by the vesicular and smooth muscle cells of the renal vessels and mediates the AVP vasopressor effects [67,83]. V1aR is also expressed in the collecting duct. Upon AVP binding, the G_q_ protein subunits dissociate and the α_q_ subunit activates phospholipase C-β (PLCβ), which increases diacyl-glycerol (DAG) and inositol triphosphate (IP3), releases calcium (Ca^2+^) from the endoplasmic reticulum and activates the transient receptor potential (TRP) ion channel, which replenishes the Ca^2+^ stores from extra-cellular Ca^2+^ [84]. V2R is expressed in the nephron, the basolateral membrane of epithelial cells of the distal convoluted tubule, and the collecting duct where it mediates the AVP antidiuretic action [24,85,86]. In the collecting duct, AVP binding to V2R dissociates the receptor from the G_s_ protein subunits (Figure 2), which activates a signal transduction cascade producing cAMP and Ca^2+^ release from ryanodine-sensitive stores [87]. Such a cascade leads to protein kinase A (PKA)-dependent phosphorylation of aquaporin 2 (AQP2) [88]. AQP2 is a water channel stored in intracellular vesicles [89]. Upon AVP signaling, AQP2 vesicles are shuttled to and fused with the apical membrane, which enhances water reabsorption (Figure 2) [90,91]. AVP can be translocated to the cytoplasm and degraded by the proteasome, which dampens signaling and results in water excretion as diluted urine [92,93,94]. 

#### 1.5.2. Heart

Myocytes of the cardiac vascular smooth muscle express V1aR [83,96]. While AVP can protect the heart from myocardial injuries [97,98], high levels of circulating AVP and V1aR overexpression have been associated with heart failure, indicating the importance of V1aR signaling strength [83]. 

#### 1.5.3. Brain 

In the brain, V1aR, V1bR, and V2R mediate adaptive behavioral responses to centrally released AVP. Typically, the brain displays species-specific AVP receptor expression patterns, with V1aR and V1bR most commonly found in the lateral cortex [67,68]. The corticotropic cells of the human anterior pituitary are rich in V1bR, which promotes adrenocorticotropic hormone (ACTH) production. ACTH stimulates the adrenal synthesis of cortisol, androgens, and aldosterone ([63,64], reviewed in [24]). Under stress, V1bR contributes to activate the hypothalamo-pituitary-adrenal axis [99]. Implicating AVP in social behavior and development, V1bR is also found in the CA2 region of the hippocampus and the anterior region of the amygdala [100]. RT-PCR analysis indicated stable *V2R* mRNA expression in the rat cerebrum and age-dependent decline in the hippocampus [55]. However, V2R function in the brain is unclear [31].

#### 1.5.4. Other Tissues 

In the human and rodent pancreas, AVP binding to V1bR augments insulin release [72,101]. Conversely, AVP engagement of hepatic V1aR increases glycemia and, when prolonged, may predispose to glucose intolerance and diabetes [102,103]. As detected by RT-PCR, the *V1bR* mRNA is also expressed in the adrenals and small intestine, albeit how these cells respond to AVP remains undefined [72].

#### 1.5.5. Regulation of AVP Receptor Expression 

Several tissue-specific signals affect AVP receptor expression transcriptionally and post-transcriptionally. In the collecting duct, metabolic acidosis increases V1aR [104] and decreases V2R expression [85]. Dehydration upregulates V2R expression [85]. V2R engagement by AVP stimulates faster ubiquitin-dependent degradation than steady-state turnover [105]. V2R expression levels are sex-dependent, which may have a bearing for AVP-V2R-linked pathologies (e.g., PKD below) [106]. In addition, both *V1bR* transcription and translation change in response to stress [107,108]. 

#### 1.5.6. Receptor Desensitization

Alike other GPCRs, the signaling cascade from AVP receptor activation also results in receptor desensitization, which interrupts downstream signaling [109,110,111]. This occurs through GPCR phosphorylation by GPCR kinases, arrestin binding, G protein uncoupling, and receptor internalization. V1aR is rapidly recycled to the cell surface; in contrast, V2R is sequestered in perinuclear recycling compartments [112,113,114]. Because AVP receptors can be phosphorylated by several kinases, heterologous desensitization can occur in response to different signals (e.g., angiotensin II) [115]. 

## 2. AVP-Related Renal Pathology

Because of its many physiological roles, AVP dysregulation due to both loss-of-function and gain-of-function leads to widespread complications. Mutations in the *AVP*, *V2*, and *AQP2* genes cause forms of diabetes insipidus that have been extensively reviewed elsewhere [6,116,117,118]. Here, we will focus on AVP gain-of-function in PKD. 

### 2.1. Polycystic Kidney Disease and AVP 

In PKD, cystic degeneration of the kidneys progressively affects their function, disrupting water balance. Autosomal dominant PKD (ADPKD) is a hereditary renal disease affecting 12.5 million people worldwide [119]. The majority of mutations found in ADPKD patients map to the *PKD1* and *PKD2* genes [119,120]. In rare autosomal-recessive PKD (ARPKD), mutations disrupt the *PKHD1* gene [119,121,122]. *PKD1*, *PKD2*, and *PKHD1* all encode transmembrane proteins, namely polycystin 1, polycystin 2, and fibrocystin [122]. Polycystin 1 is considered an orphan, atypical GPCR. Polycystin 2 is a Ca^2+^ permeable non-selective cation channel with homology to the TRP superfamily [123,124,125]. Fibrocystin, or polyductin, is also a transmembrane protein [126,127,128,129]. Polycystin 1, polycystin 2, and fibrocystin interact and can be found at the cilium, considered critical for PKD [124,125]. The precise mechanisms of cyst formation and growth are unknown; however, several changes are known to occur at the molecular, cellular, and physiological levels that affect tubular homeostasis and function. Neoplastic-like cystic growth and tubular epithelial cell apoptosis characterize PKD [119,130]. Early PKD stages are characterized by abnormally high fluid excretion, which causes dehydration and activates compensatory AVP release to stem water loss [119,130,131,132,133,134]. While in ADPKD congenital cysts only occur in 1–3% of nephrons, continued cystic growth and new cysts eventually deform and compress the surrounding parenchyma, impair nearby nephrons, and increase cell death. At advanced PKD stages, kidneys can quadruple their volume and reach the size of a football [119]. These events eventually overwhelm renal compensation, causing end-stage renal disease and kidney failure in half of the ADPKD patients [119,130]. AVP, V2R, and cAMP signaling are all altered in PKD [122,135,136,137,138]. Moreover, *AVP* loss of function in the PCK rat (*AVP*^−/−^) increases renal cAMP and ERK phosphorylation, reminiscent of ARPKD [122]. 

**Figure 3:**
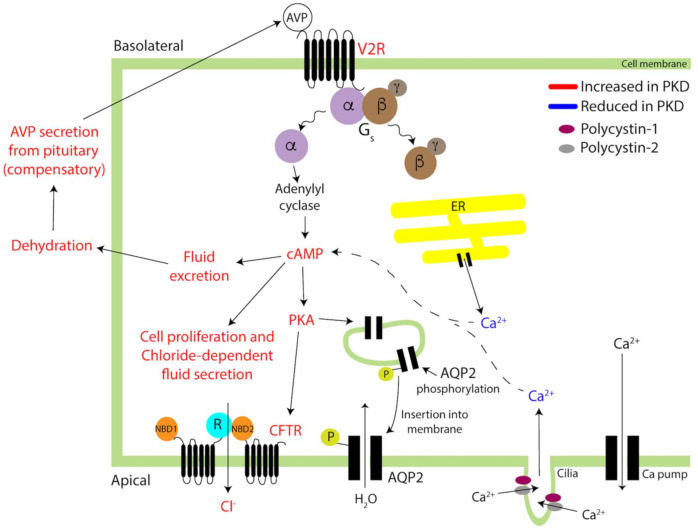
V2R and AVP signaling in normal and autosomal dominant polycystic kidney disease (ADPKD) renal tubule epithelial cells. In normal cells, AVP binding to V2R promotes dissociation of the trimeric G_s_ into its α and βγ subunits. The α subunit triggers adenylyl cyclase (AC)-mediated cAMP synthesis, which activates PKA and phosphorylates AQP2. Phospho-AQP2 is shuttled to the apical cell membrane and water reabsorption increased. In ADPKD (red type), reduced Ca^2+^ release from the ER and impaired Ca^2+^ import from polycystin 2 at the primary cilia elevate intracellular cAMP (dashed), which in turn increases fluid excretion. Resulting dehydration triggers AVP release from the pituitary. Furthermore, higher than normal intracellular cAMP in ADPKD boosts Cl^−^ transport via the cystic fibrosis transmembrane conductance regulator (CFTR) channel that contributes to cystic cell proliferation and chloride-dependent fluid secretion.

*PKD1* and *PKD2* mutations reduce Ca^2+^ signaling in the primary cilia and endoplasmic reticulum (ER) of the epithelial cells of the renal tubule, which in turn increases intracellular cAMP and fluid excretion ([133], Figure 3). Concomitant up-regulation of V2R expression in PKD kidneys further elevates intracellular cAMP, which in turn increases protein kinase A activity and Cl^-^ secretion via the cystic fibrosis transmembrane conductance regulator (CFTR), fueling cystic cellular proliferation and Cl^-^-dependent fluid secretion [131,139]. Finally, in PKD, V2R may redistribute apically in the tubular cells [137]. ARPKD cells also feature AVP/V2R upregulation, cAMP-activated cellular proliferation, and reduced intracellular Ca^2+^ [135,140,141]. Therapeutic targeting of the AVP system with V2R antagonists appears to be moderately effective in the short term and is discussed in Section 5.1.2.

### 2.2. Biological Sex and PKD 

Like female mice, women display higher *V2R* mRNA expression than their male counterparts ([106], reviewed in [142]) corroborating evidence for sex-specific phenotypic, clinical, and/or pharmacological differences in the AVP response. The relationship between sex and PKD appears complicated and is incompletely understood. Notably, while men seem more sensitive than women to several kidney diseases, in ADPKD such difference appeared to be reduced, implicating that women may instead have faster disease progression [143]. More than 80% of ADPKD and severe polycystic liver disease patients are females, suggesting that hormonal regulation may contribute to disease severity [119]. However, in a recent study, female patients displayed slower cystic progression than males, adding to a recent proposal that men may be more severely compromised by ADPKD than women, despite the latter being more affected numerically [144]. In contrast, both sexes are equally affected by ARPKD [119]. Future studies will be needed to clarify this important aspect of the PKD pathophysiology. 

## 3. AVP and Heart Failure

As of the year 2015, cardiovascular disease had caused the death of 17.3 million people worldwide, to which HF has been one of the leading causes [145]. The decline in the heart’s ability to effectively operate as a fluid pump and maintain proper systemic circulation, HF translates clinically into decreased cardiac output and stroke volume, regardless of total circulatory blood volume [146]. Chronic HF patients exhibit two to three times higher serum concentrations of AVP (2.5–6.4 pM) compared to healthy individuals (<1.6 pM). Higher AVP levels are associated with later stages of HF and suggest that AVP may contribute to disease progression [147,148]. AVP synthesis within the SON and the PVN is coordinated with the afferent signaling pathways from baroreceptors localized within the aorta, the carotids, the cardiac atria, and left ventricle [149,150]. In HF, decreased circulating blood volume reduces baropressor sensitivity and stimulates non-osmotic AVP release [146]. Moreover, as a vasoconstrictor, AVP raises blood flow. Specific to HF, systemic AVP release augments peripheral vascular resistance and compensates for reduced cardiac output and stroke volume in the short-term [149,151]. However, chronic AVP hyperstimulation eventually impairs the heart’s mechanical function, promotes extensive cardiac remodeling, and causes fluid imbalances that synergistically exacerbate cardiac dysfunction and lead to HF [147,152].

### 3.1. AVP-V1aR Signaling and Cardiac Contractility

**Figure 4:**
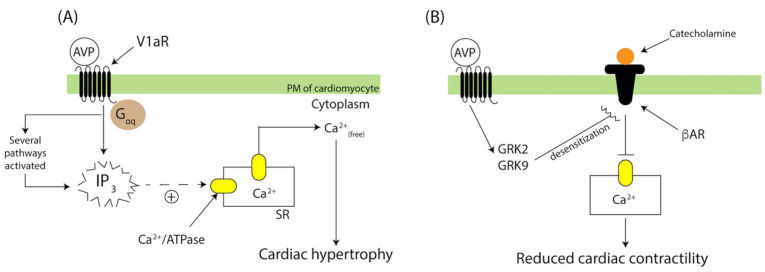
AVP-V1aR signaling contributes to weaker myocardial contractions and cardiac remodeling in heart failure (HF). (**A**) AVP binding to V1aR during HF activates G_αq_ protein-mediated signaling, which amplifies IP_3_ signaling and triggers Ca^2+^ release (dashed) from the sarcoplasmic reticulum (SR). Prolonged Ca^2+^ mobilization leads to myocardial hypertrophy. (**B**) G_αq_-independent signaling promotes GRK recruitment to the plasma membrane (PM) decreasing catecholamine-β adrenergic receptor (βAR) activation and Ca^2+^ mobilization. When prolonged, this condition impairs myocardial contractions.

In HF patients, elevated plasmatic AVP appears to correlate with *V1aR* mRNA upregulation in the left ventricular myocardium [98]. Similarly, HF induced by left coronary ligation in the *Ntac:SD*^+/+^ rat also featured upregulated *V1aR* mRNA within the left ventricle [153]. Probing of the AVP-V1aR signal transduction pathway revealed that overexpression of the human V1aR in mice cardiac myocytes, (*V1a*^tTa/+^) augmented AVP-V1aR signaling and promoted G_αq_ protein recruitment to the plasma membrane, increased D-myo-inositol 1,4,5 trisphosphate signaling and Ca^2+^ mobilization (Figure 4A). V1aR overexpression leads to extensive myocardial contraction, hypertrophy, vasoconstriction, and reduced cardiac contractility [154,155]. Persistent AVP-V1aR signaling also disrupts β-adrenergic receptor activation and signaling (Figure 4B) [156]. Hence, physiological coupling of higher AVP secretion and V1aR density appears to underlie changes in cardiac contractility and morphology in HF.

### 3.2. AVP-V1aR Signaling and Cardiac Remodeling 

Cardiac fibroblasts are non-contractile cells amounting to up to 60% of the heart, essential to maintain the structural integrity of the heart’s extracellular matrix (ECM) [152]. Activation of V1aR signaling was found to promote cardiac fibroblast proliferation and function [147,152]. In cardiac fibroblasts, AVP-V1aR dependent signaling recruits GRK2 and β-arrestin1/2 and increases expression of matrix metalloproteinases MMP2 and MMP9, functioning in ECM degradation and tissue remodeling [147,157]. Moreover, AVP-V1aR signaling induces phosphorylation of mitogen-activated ERK1/2 kinase that binds GRK2 and β-arrestin1/2 and stimulates cardiac fibroblasts proliferation [151,157]. Extensive ECM deposition follows increased expression of connective tissue growth factor and endothelin-1 that respectively promote collagen synthesis and inhibit MMP1 [147]. The combined vasopressor function of AVP and endothelin-1 increase peripheral vascular resistance and cardiac afterload, which—when prolonged—lead to adaptive myocardial hypertrophy to try maintaining cardiac output to the periphery [146,149]. 

### 3.3. AVP-V2R Signaling for Fluid Volume Retention and Cardiac Function 

In a healthy individual, the baroreceptor-mediated non-osmotic release of AVP is meant to limit perturbation from normonatremic and euvolemic states and ensure proper cardiac compliance. However, in the case of progressive HF, sustained non-osmotic AVP release will induce AQP2-mediated water reabsorption at renal collecting ducts and cause dilutional hyponatremia (serum Na^+^ level <134mEq/L) and expansion of the circulatory blood volume [158,159]. A coronary-ligated rat model of congestive HF indicated that AQP2 was also upregulated and further impaired cardiac performance through greater infarction of the left ventricular free wall [160,161]. Moreover, prolonged AQP2 translocation to the apical membrane increased systemic fluid retention and fluid saturation/hypervolemic state and cardiac preload. If sustained, this condition stresses the diastolic wall, and promotes MMP activation and cardiac hypertrophy [147]. Altogether, chronic AVP secretion coupled with activation of V1aR and V2R signaling contribute to a vicious cycle of extensive myocardial remodeling and inefficient contractile events that usually escalate into fatal HF [160,162]. 

## 4. AVP and Brain Function

AVP functions as a neurohormone regulating memory and attention [163,164], increases neural transmission in the amygdala [165,166] and participates in the neuroendocrine stress response [167]. AVP modulates social behavior in several fish, amphibian, vertebrate, and mammalian species with sexual dimorphic displays ([168,169,170], reviewed in [31]). 

The AVP-synthesizing magnocellular neurons of the PVN project to the posterior pituitary and release AVP into the circulatory system to induce water retention [171,172,173,174]. In contrast, the parvocellular neurons project from the PVN to the median hypothalamic eminence, and release AVP to trigger the secretion of ACTH and several anterior pituitary hormones [172,175]. Other AVP-synthesizing neurons can be found in the medial amygdala, the bed nucleus of the stria terminalis (BNST), and the SCN, which all project centrally to the brain preoptic and olfactory areas, hypothalamic, and extra-hypothalamic regions [54,176,177,178]. These neurons are thought to be the source of neural AVP because the blood brain barrier is impermeable to plasmatic AVP [166,179,180,181]. Seven of the nine amino acids in AVP are identical to OT, another neurohormone, due to the partial overlap of their genes [182,183]. The distinct expression and distribution of AVP, OT, their receptors, and the reach of their neuronal projections further intertwine these systems (reviewed in [68,166]). AVP and OT can function variably and antagonistically, depending on sex and several context-dependent factors. While AVP appears anxiogenic, OT is anxiolytic and pro-social [33,181,184,185,186,187]. Recently, it was found that the similarity between OT and AVP results in their binding to each other’s receptors and substantial crosstalk in vivo, especially at high AVP and OT concentrations [27]. Integrative functional models of AVP and OT signaling are needed to reconcile observations made in disparate experimental set ups and behavioral paradigms, with primary focus on OT and male individuals, to refine understanding of the neural circuitry regulating social behaviors. Here, we will examine the AVP functions, referring readers interested in the biology of OT to comprehensive reviews [163,166].

### 4.1. AVP and Animal Behavior 

AVP and its non-arginine vasopressin relatives (VP) preside to sociality in several rodents (rats, mice, hamsters, voles, jerboas) as well as birds and fish through largely conserved neural networks in the amygdala, BNST, lateral septum, medial preoptic area, anterior hypothalamus, and the periaqueductal grey [68,169,188,189,190,191,192,193,194,195,196]. Despite these similarities, the behavioral responses appear both species-specific and may vary among conspecifics, likely due to the differential distribution and expression of neural AVP receptors and influence by gonadal hormones [169,177,196]. Note, V1aR and V1bR are widely expressed in the brain (Table 1). To probe the AVP neural functions in vivo, gene knock-out of *AVP* or its receptors and pharmacological targeting of AVP receptors have been employed in combination with behavioral tests such as the forced swim test, which assesses the animal’s overwhelm from a prolonged drowning threat and the elevated plus maze test that measures anxiety ([43], reviewed in [197]). In such contexts, the AVP response appeared largely subject-specific and influenced by social experience, hormonal status, and the neuronal connections within the hypothalamus and between the hypothalamus and other areas [178,198,199]. 

#### 4.1.1. V1bR and Behavior 

V1bR engagement by AVP in the anterior pituitary and adrenal medulla promotes the release of key stress hormone ACTH [25,200,201]. This adds to the V1aR-mediated synthesis and cortisol release occurring in the adrenals [70]. Denoting altered stress response, during the forced swim test and elevated plus maze, male *V1bR*^−/−^ mice displayed reduced resting levels and impaired ACTH release compared to controls [25,99]. V1bR may also mediate aggression [202,203], and the *V1bR*^−/−^ mice display social deficits [204,205]. 

#### 4.1.2. V1aR and Behavior

V1aR may regulate individual recognition, pair-bonding, sexual behavior, social memory, and aspects of parental care (e.g., maternal aggressive behavior, anxiety, depression) [25,169,206,207,208,209]. Intracerebral AVP microinjection and V1aR overexpression in the lateral septum both improved social recognition; conversely, both *V1aR* knockout and administration of V1aR antagonists severely impaired sociality [208,210,211,212]. 

#### 4.1.3. Recognition 

In rodents, species-specific recognition is largely based on olfactory cues, and depends on species-specific distribution of AVP-immunoreactive nerve fiber types [213]. Pharmacological or genetic V1aR targeting prevented short-term social recognition in rats, possibly through changes in olfactory processing [178,214,215].

#### 4.1.4. Aggression

Several AVP-immunoreactive neurons in the medial amygdala and BNST that project to the lateral septum and AVP-immunoreactive projections to the anterior hypothalamus were implicated in aggression in rodents [169,216,217,218,219]. While this theme is conserved in several species, anatomical specializations are thought to underlie species-specific behaviors. For example, VP infusion into the septum inhibited aggression against intruders in the field sparrow, a territorial species [217], whereas it increased aggression in the colonial zebra finch [218]. Note, the septum is involved in social cognition, stress response, and anxiety. 

#### 4.1.5. Parental Care 

In rodents, parental care (e.g., pup licking and grooming) is necessary for both early development and the adoption of similar nurturing behavior as adults. Genetic and pharmacologic evidence indicated that in both sexes, pup grooming and maternal post-partum aggression are mediated by the AVP/VP network and the neuronal connections between the hippocampus and both the amygdala and the basal forebrain (reviewed in [81]). Engaging in parental behavior increased expression of both AVP and its receptors, which consolidates gene expression and behavior [209,220,221,222,223]. 

#### 4.1.6. Sexual Behaviors

The effects of AVP/VP on sexual behavior have been thoroughly reviewed elsewhere [224].

#### 4.1.7. Differential Sex Response to AVP 

In most species (except hyenas and rats), estrogen and androgens seem to increase AVP levels [177]. In rats, testosterone was found to modulate AVP/VP receptor expression and localization, as well as the number of AVP-immunoreactive neurons [225,226,227,228]. Conversely, castration reduced AVP expression in a subpopulation of cell bodies within the BNST of amphibians, birds, and mammals [148,229]. Note, the BNST is a sexually dimorphic center that integrates limbic information and valence monitoring and has been implicated in several psychiatric disorders. Corroborating the importance of AVP signaling strength, compared to females, male rats showed denser AVP-immunoreactive fibers in the lateral septum and lateral habenular [230,231,232] that may impact a subset of AVP/VP responses ([233], reviewed in [31]). In naked mole rats, breeding dominant males and females were found to contain more AVP-immunoreactive neurons than subordinates [177,234]. In mandarin voles, dominant and subordinate females displayed different distributions of AVP-immunoreactive neurons in the PVN and SON [235]. 

#### 4.1.8. Changes in AVP-Signaling 

The AVP system appears to adaptively respond to conditions and environments. For example, maternal stress during late pregnancy reduced *V1aR* neural expression and impaired pup sociality [236]. Seasonal breeding [237,238] and fatherhood [239,240,241,242] also appear to modulate AVP signaling. 

#### 4.1.9. Circadian Response 

V1aR signaling affects circadian rhythmicity [71,243]. In mice, circadian non-endocrine regulation elicited by SCN neurons triggered AVP release, OVLT activation, and increased thirst before sleep to counter dehydration during the night hours [244,245].

### 4.2. AVP and Human Behaviour

The AVP (and OT) systems underlie human social cognition, with short-term effects on context-specific behavioral responses and long-term behavioral regulation (e.g., anxiety, reward) [246,247,248]. Alike other species, OT is pro-social [148,249,250,251,252]. Depending on context, AVP can promote either pair bonding and cooperation or threat reaction and anxiety [253,254,255]. Patients with certain personality disorders presented high AVP in the cerebrospinal fluid, and often systemically [174,256]. Autism spectrum disorder (ASD), Williams syndrome, schizophrenia, depression, social anxiety, and attachment disorders all respond to AVP receptor blockade or seem linked to AVP and OT [166,257,258,259,260,261,262,263,264,265,266,267,268]. Reduced AVP was found in patients with schizophrenia and bipolar disease, with lower AVP levels possibly predisposing to psychoses, independent of OT [264,269]. Social stress is known to increase odds to develop psychiatric disorders [270], albeit its effects on AVP are unknown. Suggesting that AVP signaling may interface with the limbic system, serotonin modulates AVP release and V1aR activation [271,272]. Certain polymorphic variants of *V1aR* and the serotonin transporter *SLC6A4* that are linked to creative and behavioral traits have been found frequently associated in dancers and may relate to capacity for social empathy [273]. Depression, impulsivity, and violence seem causally linked to AVP regardless of other factors, e.g., sex, stressors [274,275,276,277]. Childhood stress appears to affect sociality through AVP [278]. Underscoring the complexity of the AVP neural network, the AVP system was found to respond to both gonadal steroids and genetic background (more below), and life history influences the individual responses to specific contexts [67]. Possibly contributing to physiological and individual diversity, sensitivity to OT signaling and its AVP-balancing functions seem to be established during early life [279]. Moreover, the AVP-responsive amygdala, cingulate gyrus, and hypothalamus all impinge on social behavior [261,280,281,282]. Recently, AVP-immunoreactive projections have been found in the human male agranular insula [282], a region of the brain neocortex presiding to sensory processing, high-level cognition, and affection [283]. Thus, AVP appears to be central to the human neural developmental pathways. Polymorphic microsatellites upstream of the *V1aR* gene may influence expression levels in the amygdala [284,285,286,287,288]. Furthermore, some *V1aR* gene polymorphisms associate with behavioral traits, including novelty seeking, sexual behaviors, musicality, and dance ([261,273,289,290], reviewed in [166]). Specific polymorphisms and simple sequence repeats in the *V1aR* gene associate with schizophrenia and ASD, social cognition deficits [34,291], and behaviors including aggression, altruism, depression, and empathy [269,292,293]. Polymorphisms reducing *AVP* gene expression have been linked to schizophrenia [294]. Consistent with a causal relationship, AVP administration lessened the negative symptoms of some schizophrenic patients [264]. 

To study the effects of AVP in human subjects, intra-nasal administration is used in combination with functional MRI (fMRI) to reveal the brain areas with changes in deoxyhemoglobin concentration in response to AVP [33,178,295,296]. As in animals, intranasal AVP administration may increase endocrine stress by augmenting amygdala activity [165] and promoting ACTH secretion [167] and favor negative responses in ambiguous social situations [297]. While AVP reduced friendliness in men, it increased it in women [298]. Brain fMRI during a Prisoner’s Dilemma Game revealed that, upon intranasal administration of 20 international units (IU) of AVP and compared to placebo, AVP increased BNST activity and reciprocated cooperation in men [255,299]. Note, the AVP and OT-responsive BNST interfaces with the brain areas regulating affiliation, parental care, sexual behaviors, communication, and aggression [190,300]. In contrast, in women, AVP activated the left caudate nucleus and left amygdala and increased cooperation following partner defection [299]. In men, perceived unreciprocated cooperation appeared to reduce the activation of the right amygdala, which is responsible for processing negative emotions, and the anterior insula [148]. Interestingly, these patterns may be influenced by personality traits. Upon AVP administration, male participants with high neuroticism score also displayed higher activity in the anterior cingulate cortex, medial prefrontal cortex, and lateral temporal lobe in response to unreciprocated cooperation [301]. Note, such regions mediate emotion, decision-making, and language. Conversely, cooperation activated the right insula, which is linked to body awareness [301]. AVP increased male cooperation in risky yet rewarding choices through decreased activity of the left dorsolateral prefrontal cortex (which modulates cognitive flexibility during risky cooperation) [254]. AVP may also alter male emotional processing. Normally, seeing facial displays of fear or anger changes activity in the subgenual cingulate of the medial prefrontal cortex, which is part of the limbic system. However, men treated with 40 IU) of intranasal AVP neither recognized the difference between such displays, nor showed differential brain activity, and exhibited decreased functional connectivity between the subgenual and supragenual cingulate [262]. Thus, the human AVP response seemingly shares traits with other mammals and birds, and also displays unique species- and individual-specific traits due to individual genetics, life history, and situation, which all contribute to the integration of sensory processing and behavior to adapt to specific contexts. 

## 5. Pharmacological Modulation of the AVP Pathways

The human AVP pathway has been targeted pharmacologically using AVP receptor agonists and antagonists. Agonists are mainly AVP analogs that bind to the same receptor site (orthosteric) as natural AVP, which are used to bolster AVP signaling in loss-of-function conditions, e.g., diabetes insipidus and septic shock ([302], reviewed in [303]). Conversely, antagonists lessen elevated AVP signaling (gain-of-function) in HF, PKD, and secondary shock, and are aquaretic, i.e., promote excretion of solute-free water (reviewed in [304]).

### 5.1. Vasopressin Receptor Antagonists 

Known also as vaptans, nonpeptide AVP receptor antagonists are administered orally or intravenously to bind specifically and competitively to renal and cardiac V2R [305,306]. Tolvaptan, lixivaptan, satavaptan, and mozavaptan each have distinct chemical features that uniquely influence receptor affinity and biological activity (Table 2). Upon receptor interaction, the vaptans prevent AQP2 translocation to the apical membrane of the renal collecting duct and increase aquaresis [307,308,309]. Conivaptan exhibits high affinity for both V1aR and V2R. Upon binding to localized V1aRs, conivaptan can limit myocardial hypertrophy [310,311]. Balovaptan selectively binds to neural V1aRs and exhibits potential for treating human behavioral disorders [312,313]. Relcovaptan similarly binds to V1aRs and is used to induce local vasodilatation in Raynaud’s disease and reduce preterm labor contractions [314,315]. Note, Raynaud’s disease causes vasoconstriction in hands and feet, and may lead to localized tissue necrosis. Nelivaptan interacts with neuronal V1bRs, reduces ACTH secretion by approximately 15%, and is a potential anxiolytic and antidepressant [64,316,317]. Nelivaptan may also function in other tissue contexts. Treatment of rodent and human pancreatic β-cell isolates eliminated insulin release [101]. Moreover, V1bR-selective non-peptide antagonist SSR-149415 abolished Ca^2+^ release, glucagon secretion, and cell proliferation of a glucagon-secreting α-pancreatic cell line [72]. Non-competitive AVP receptor antagonists may affect downstream receptor signaling independent of AVP binding and may offer functional selectivity, i.e., modulation of only a subset of a receptor’s downstream signaling pathways. Preserving some of the wanted responses induced by binding of the natural ligand, such molecules may reduce the side effects caused by receptor blockade. While this is an untapped potential in vasopressin research, one such compound, the VRQ397 peptide, appeared specific for V2R, behaved as an allosteric modulator, and exhibited aquaretic properties [318]. 

#### 5.1.1. V2R Antagonism in Heart Disease Therapy

Because of their aquaretic effects, the vaptans are used in patients with congestive HF with simultaneous hypervolemic hyponatremia and edema [305,341]. The inhibitory effect of vaptans on the V2R signaling cascade prevents the translocation of AQP2 channels to the apical membrane of the renal collecting duct cells. The ensuing aquaresis corrects existing fluid and/or electrolytic imbalances [158]. Administration of 30–45 mg of tolvaptan can reduce edema, body weight, and serum sodium level without compromising blood pressure and renal function [306].

#### 5.1.2. V2R Antagonism in PKD Therapy

Based on the strong AVP deregulation observed in ADPKD and ARPKD animal models, strategic reduction of AVP-mediated signaling and intracellular cAMP using V2R antagonists was found to reduce both cystic fluid secretion and cyst size in murine PKD models [122,342,343] and PKD patients [130,136,321]. The V2R antagonist and benzazepine derivative OPC31260 [338] reduced cystic volume and inhibited new cyst formation in several rodent PKD models [122,342] and nephronophthisis, another renal cystic disease [344]. With its high affinity for V2R, OPC31260 can displace AVP from V2R and V1aR (IC50, respectively 1.4 × 10^−8^ M and 1.2 × 10^−6^ M) [338]. In the ADPKD model of the *Pkd*^−/tm1Som^ mice, OPC31260 lowered renal cAMP, AQP2, and V2R expression (all higher than normal in ADPKD) and reduced kidney weight to wild-type levels [342]. OPC31260 also improved the PCK *AVP*^−/−^ ARPKD rat, reducing both cAMP and kidney-specific ERK phosphorylation, and overall improving water reabsorption, reducing kidney cysts and weight compared to vehicle-treated controls [122]. Due to these early encouraging results, tolvaptan, which was already approved to treat heart disease in several countries, was entered in clinical trials to normalize V2R signaling in PKD.

#### 5.1.3. Clinical Trials

Tolvaptan inhibited cystic cell proliferation by reducing activation of the B-Raf/MEK/ERK pathway and Cl^−^ secretion [136]. Several clinical trials are listed in the clinical trials databases for tolvaptan, other vaptans, and AVP agonists (www.clinicaltrials.gov, www.clinicaltrialregister.eu, Supplementary Table S1). The phase 3 TEMPO trial included 1445 patients between the ages of 18 and 50, who had a kidney volume of at least 750 mL (i.e., 50% increase compared to healthy subjects) and a creatinine clearance of at least 60 mL/min (i.e., moderately declined renal function) [321]. During a three-year period, tolvaptan treatment almost halved kidney growth and slowed kidney functional deterioration by a slope of −2.61 (mg/mL)^−1^ per year, compared with −3.81 (mg/mL)^−1^ from placebo [321,345]. These exciting results were tempered by adverse effects observed in the tolvaptan group as opposed to placebo. Despite adoption of split-dose protocols that reduce nightly excretion, uncomfortable aquaresis challenged patient compliance. Importantly, troubling unrelated hepatotoxicity raised concerns for the safety of long-term use [321,345,346]. A recent one-year trial, REPRISE, involved 1370 patients aged 18 to 55 with a GFR of 25 to 65 mL/min/1.73 m^2^, and patients aged 56 to 65 with an associated GFR of 25 to 44 mL/min/1.73 m^2^ (i.e., patients with CKD stage G3 and impaired kidney function) [130]. In this follow-up trial, tolvaptan similarly retarded the loss of renal function for advanced PKD patients, but elevated hepatic enzymes (e.g., alanine aminotransferase) to the same levels observed in chronic hepatitis, although levels renormalized upon treatment termination [130]. Tolvaptan is recommended to patients with the following characteristics: 1—age 18–50 years old, 2—fast disease progression, 3—low water and/or high salt intake [347]. Tolvaptan effectiveness may attenuate over time [321]. Importantly, none of the V2R antagonists affected liver cysts (frequent in ADPKD patients) because hepatocytes do not express V2R [348]. Currently, tolvaptan represents the only approved therapy available to (a subset of) ADPKD patients. Further studies are ongoing (Supplementary Table S1) and required to clarify its effects and applicability, as well as explore the properties of other prospective V2R antagonists that could potentially benefit a wider group of ADPKD patients.

Balovaptan was used in Improve Social Communication in Autism (VANILLA), a phase 2 clinical trial of 223 individuals with ASD. Doses of 4mg and 10mg of balovaptan improved the score of the Vineland-II Adaptive Behavior Scale of ASD patients with few adverse side effects [312]. Thus, balovaptan may be a prospective alternative to antipsychotics, that were instead found to increase risk of obesity, type 2 diabetes, and cardiovascular disease [349].

### 5.2. Synthetic AVP Analogs

Several AVP synthetic analogs (Table 3) are used in therapy or are undergoing clinical trials for conditions with reduced AVP signaling (Supplementary Table S1).

## 6. Conclusions

Conserved among the terrestrial vertebrates, vasopressins are a group of nonapeptide hormones central to maintain homeostasis and adapt to environmental and social changes in shared, species-specific, and individual ways. Characterized by an arginine in position 8, the human AVP has been studied for its effects on the kidney, heart, and brain physiology. Several cell types produce AVP and release it in the blood stream to affect water re-adsorption in the terminal region of the renal tubule, and vasoconstriction to conserve water and facilitate the cardiovascular function. AVP also functions as an endocrine modulator that affects leukocytes and immunity and the insulin/glucagon pathways. Because of the blood brain barrier impermeability, AVP is released centrally in the brain. The distribution and localization patterns of AVP-immunoreactive cells display sex- and individual specificity. New AVP-immunoreactive structures are expected to be revealed by neuroimaging advances. Widespread and regulated expression of three main high-affinity AVP receptors, V1aR, V1bR, V2, and at least one other receptor, OTR, that can be cross-activated at high AVP concentrations, indicates that many cell types can respond to AVP signaling, depending on context, which is consequential for pharmacological intervention.

While the fluid homeostasis and pressor functions of AVP are believed to have enabled colonization of a terrestrial environment, neural AVP appears to be equally important for survival in that it coordinates sensory processing and behavioral modulation through the intricate connections of AVP-immunoreactive neurons in distinct brain areas. Some AVP-dependent pathways are sensitive to gonadal steroids, serotonin, OT, and possibly other hormones, which enables adaptive fine-tuning of the AVP-dependent physiology to the changing needs of a growing individual (e.g., sexual behavior, reproduction, seasonal adaptations), and sociality (e.g., individual recognition, emotional processing, social stress, communication, parental behavior).

Altered AVP signaling has been linked to diabetes insipidus, PKD, HF, and psychiatric conditions including ASD, bipolar, and borderline personality disorder. Thus, modulators of AVP physiology have pharmacological interest. Receptor antagonists with distinct selectivity, affinity, and half-life in vivo have been identified and are being used to modulate branches of the AVP physiological responses in cardiac and renal disease, as well as to relieve neurological symptoms and ameliorate the social difficulties in borderline personality disorder. Deciphering the mechanistic aspects of AVP signaling both in tissue- and cell-specific detail, their crosstalk and systemic integration is therefore fundamental to understand how AVP adaptively integrates physiology and behavior and develop safe and effective therapeutics to treat AVP-related conditions including PKD, HF, and ASD.

## Figures and Tables

**Table 1:** AVP receptor function and expression.

Target Tissue	Receptor	Function	References
Kidney—Macula densa, intermediate, distal and collector tubules	V2R	Signal transduction, AQP2 shuttling to cell surface and water permeability, *AQP2* mRNA synthesis, intracellular cAMP regulation	[14,53,54,55,56]
Kidney—Mesangial cells, efferent arterioles, renal tubules	V1aR	Vasoconstriction	[57,58,59]
Kidney	V1bR	Unknown	[16,60,61]
Vascular Smooth Muscle	V1aR	Vasoconstriction, myocardial hypertrophy, *V1aR* mRNA upregulation, hypertension	[53,54,58,62]
	V2R	Vasodilation	[53]
Brain—Anterior Pituitary	V1bR	ACTH secretion, stimulation of endocrine response to stress	[17,53,63,64,65]
Brain—diffused expression	V1aR	Regulation of emotional and adaptive behaviors, pain	[66,67,68,69]
Brain—HPA axis (adrenal cortex)	V1aR	Cortisol synthesis and secretion	[70]
Brain—SCN	V1aR	Circadian rhythm	[71]
Brain—Cerebellum (rats)	V2R	Unknown	[55]
Pancreas	V1bR	Glucagon release, intracellular Ca^2+^ regulation, cell proliferation	[54,72]
Liver—Hepatocytes	V1aR	Glycogenolysis	[18,53]
Blood—Platelets	V1aR	Platelet aggregation	[53,73]
Blood—Leukocytes	V1aR	Chemotaxis, chemokine and antibody production	[74,75]
Myometrium	V1aR	Uterine contraction	[53,76]
Prostate	V1aR	Unknown, causative upregulation in castration resistant prostate cancer	[18,77]
Skeletal muscle	V1aR	Unknown	[18]
Lung	V1aR V2R	Unknown Anti-inflammatory	[58] [78]
Bone	V2R	Bone remodeling	[79]
Cervical ganglion (rat)	V1aR	Unknown	[80,81]
Spleen (rat)	V1aR	Unknown	[81]
Gonads (rat)	V1aR	Unknown	[80,82]

HPA: hypothalamic–pituitary–adrenal, ACTH: adrenocorticotropic hormone, SCN: suprachiasmatic nucleus.

**Table 2:** AVP receptor antagonists.

AVP Receptor Antagonists	Chemistry	Binding Affinity & MoA	Route of Administration, Dosage & Physiology	Reference(s)
**Tolvaptan** 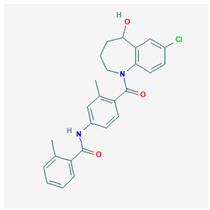	Empirical formula: C_26_H_25_ClN_3_O_2_Chemical nomenclature: *N*[4 -(7-chloro-5-hydroxy-2,3,4,5-tetrahydro-1-benzazepine-1-carbonyl)-3-methylphenyl]-2-methylbenzamide	Binds V2R (pKi: 8.9–9.4) with 30-fold higher affinity than V1aR in vivo. Reduces water reabsorption (renal collecting ducts), promotes aquaresis.	Oral 15–60 mg/day Reduced rate of renal function decline, edema, body weight and serum Na^2+^ levels.	[130,306,308,311,319,320,321,322]
**Lixivaptan** 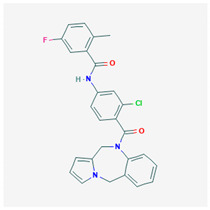	Empirical formula: C_27_H_21_ClFN_3_O_2_ Chemical nomenclature: *N-*[3-chloro-4-(6,11-dihydropyrrolo[2,1-c][1,4]benzodiazepine-5-carbonyl)phenyl]-5-fluoro-2-methylbenzamide	Binds V2R (pKi: 8.9–9.2) with 100-fold higher affinity than V1aR in vivo. Prevents translocation and localization of AQP2 to renal collecting ducts, promotes aquaresis.	Oral 30–150 mg/day or 1–10 mg/kg	[309,311,323,324]
**Satavaptan** 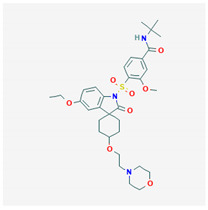	Empirical formula: C_32_H_26_N_4_O_2_ Chemical nomenclature: *N*-*tert*-butyl-4-[5′-ethoxy-4-(2-morpholin-4ylethoxy)-2′oxospiro [cyclohexane-1,3′-indole]-1′-yl]sulfonyl-3-methoxybenzamide	Binds V2R (pKi: 8.4–9.3) with 112-fold greater affinity than V1aR in vivo. Aquaretic, similar to tolvaptan.	Oral 5–25 mg/day	[307,311,325,326,327,328,329]
**Conivaptan** 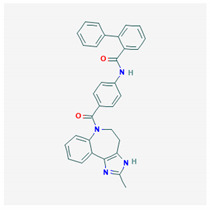	Empirical formula: C_33_H_45_N_3_O_8_S Chemical nomenclature: *N*-[4-(2-methyl-4,5-dihydro-3*H*-imidazo [4,5-d][1]benzazepine-6-carbonyl)phenyl]-2-phenylbenzamide	High affinity for both V1aR (pKi: 9.37) and V2R (pKi: 9.44) in vivo. V1aR: reduces Ca^2+^ mobilization and kinase activity (cardiac tissue), reduces myocardium hypertrophy. V2R: promotes aquaresis similar to tolvaptan.	Oral & intravenous 20–40 mg/day or 0.003–0.1 mg/kg	[310,311,322,330,331,332]
**Nelivaptan** 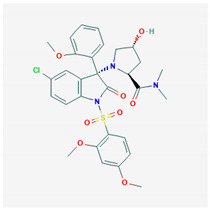	Empirical formula: C_30_H_32_ClN_3_O_8_S Chemical nomenclature: (2*S*,4*R*)-1-[(3*R*)-5-chloro-1-(2,4-dimethoxyphenyl) sulfonyl-3-(2-methoxyphenyl)-2-oxoindol-3-yl]-4-hydroxy-*N*,*N*-dimethylpyrrolidine-2-carboxamide	Binding affinity for V1bR (pKi: 5.9) in vivo. Normalizes ACTH hypersecretion in response to stress stimuli.	Oral 3–30 mg/day Potential anxiolytic and anti-depressant.	[64,316,317,333]
**Balovaptan** 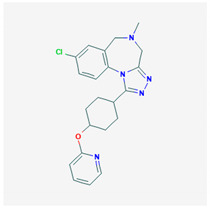	Empirical formula: C_22_H_24_ClN_5_O Chemical nomenclature: 8-chloro-5-methyl-1-(4-pyridin-2-yloxycyclohexyl)-4,6-dihydro-[1,2,4] triazolo[4,3a][1,4]benzodiazepine	Binding affinity for V1aR (pKd: 5.0, Ki: 1nM) in vivo.	Oral 10 mg/day Improved Vineland II adaptive behavior scales.	[312,313,334]
**Relcovaptan** 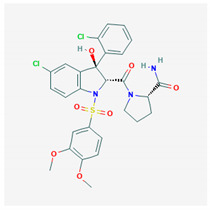	Empirical formula: C_28_H_27_Cl_2_N_5_O_7_S Chemical nomenclature: (2*S*)-1-[(2*R*,3*S*)-5-chloro -3-(2-chlorophenyl)-1-(3,4-dimethoxyphenyl) sulfonyl-3-hydroxy-2*H*-indole-2-carbonyl] pyrrolidine-2-carboxamide	Binding affinity for V1aR (pKi: 8.1) in vivo. Promotes vasodilation (vascular bed).	Oral 400 mg/day Potential for treatment of Raynaud’s syndrome and regulation of uterine contractions in preterm labor.	[314,315,335,336,337]
**Mozavaptan** 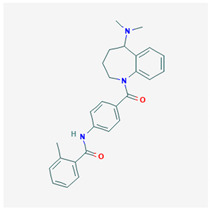	Empirical formula: C_27_H_29_N_3_O_2_ Chemical nomenclature: *N*-[4-[5-(dimethylamino)-2,3,4,5-tetrahydro-1-benzazepine-1-carbonyl]phenyl]-2-methylbenzamide	Binding affinity for V2R (pKi: 8.03) in vivo. Aquaretic.	Oral 30 mg/day or 1–30 mg/kg	[315,322,338,339,340]

Chemical structures retrieved from PubChem NIH.

**Table 3:** AVP synthetic analogs.

AVP & Synthetic Analogs	Chemistry	Binding Affinity & MoA	Route of Administration, Dosage & Physiology	Reference(s)
**AVP** 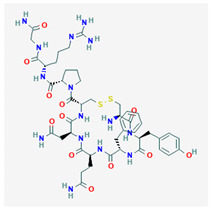	Empirical formula: C_44_H_61_N_13_O_12_S_2_ Chemical nomenclature: (2*S*)-1-[(4*R*,7*S*,10*S*,13*S*,16*S*,19*R*)-19-amino-7-(2-amino-2-oxoethyl)-10-(3-amino-3-oxopropyl)-13-benzyl-16-[(4-hydroxyphenyl)methyl]-6,9,12,15,18-pentaoxo-1,2-dithia-5,8,11,14,17-pentazacycloicosane-4-carbonyl]-*N*-[(2*S*)-1-[(2-amino-2-oxoethyl)amino]-5-(diaminomethylideneamino)-1-oxopentan-2-yl]pyrrolidine-2-carboxamide	Binds V1aR (pKi: 9.59), V1bR (pKi: 9.31) and V2R (pKi: 8.92) with high affinity. MoA: See text for details.	Oral, intravenous & intranasal 24–32 IU/day	[322,350]
**Desmopressin** 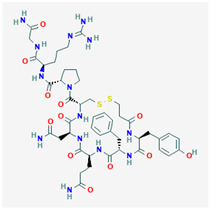	Empirical formula: C_46_H_64_N_14_O_12_S_2_ Chemical nomenclature: (2*S*)-*N*-[(2*R*)-1-[(2-amino-2-oxoethyl)amino]-5-(diaminomethylideneamino)-1-oxopentan-2-yl]-1-[(4*R*,7*S*,10*S*,13*S*,16*S*)-7-(2-amino-2-oxoethyl)-10-(3-amino-3-oxopropyl)-13-benzyl-16-[(4-hydroxyphenyl)methyl]-6,9,12,15,18-pentaoxo-1,2-dithia-5,8,11,14,17-pentazacycloicosane-4-carbonyl]pyrrolidine-2-carboxamide	Binding affinity for V2R (pKi: 8.3). Induces AQP2 apical translo-cation (renal collecting duct) and water reabsorption.	Oral & intranasal 0.2–0.6 mg/day Need frequent medical follow-up.	[322,339,351,352]
**Terlipressin** 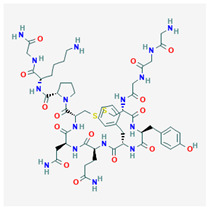	Empirical formula: C_52_H_74_N_16_O_15_S_2_ Chemical nomenclature: (2*S*)-1-[(4*R*,7*S*,10*S*,13*S*,16*S*,19*R*)-19-[[2-[[2-[(2-aminoacetyl)amino]acetyl]amino]acetyl]amino]-7-(2-amino-2-oxoethyl)-10-(3-amino-3-oxopropyl)-13-benzyl-16-[(4-hydroxyphenyl)methyl]-6,9,12,15,18-pentaoxo-1,2-dithia-5,8,11,14,17-pentazacycloicosane-4-carbonyl]-*N*-[(2*S*)-6-amino-1-[(2-amino-2-oxoethyl)amino]-1-oxohexan-2-yl]pyrrolidine-2-carboxamide	Binding affinity for both V1aR (1.1 × 10^−6^ Ki moles) and V2R (6.9 × 10^−6^ Ki moles). V1aR: induces splanchnic and renal vasocon-striction, reduces portal pressure. V2R: increases apical AQP2 and water retention.	Intravenous 1–2 mg/day as needed. Increased mean arterial pressure, decreased heart rate and improved renal function.	[353,354,355,356]
**Felypressin** 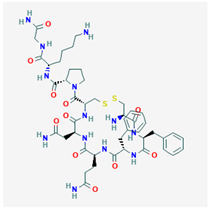	Empirical formula: C_46_H_65_N_13_O_11_S_2_ Chemical nomenclature: (2*S*)-*N*-[(2*S*)-6-amino-1-[(2-amino-2-oxoethyl)amino]-1-oxohexan-2-yl]-1-[(4*R*,7*S*,10*S*,13*S*,16*S*,19*R*)-19-amino-7-(2-amino-2-oxoethyl)-10-(3-amino-3-oxopropyl)-13,16-dibenzyl-6,9,12,15,18-pentaoxo-1,2-dithia-5,8,11,14,17-pentazacycloicosane-4-carbonyl]pyrrolidine-2-carboxamide	Binding affinity for V1aR (21 units/mg). Induces smooth muscle contraction (vascular bed).	Intramuscular 240 ng/kg Less antidiuretic effects than AVP.	[357,358,359,360]

Chemical structures retrieved from PubChem NIH.

## Data Availability

Not applicable.

## Supplementary Materials

The following are available online at https://www.mdpi.com/2227-9059/9/1/89/s1, Table S1: Clinical trials.
