# Supplementary material for: The Biology of Vasopressin
Source: Biomedicines. 2021 Jan 18;9(1):89. doi: 10.3390/biomedicines9010089 (PMC7832310; doi:10.3390/biomedicines9010089)
Supplement: Supplementary file 1 [file biomedicines-09-00089-s001.pdf]

Table S1: Clinical trials of AVP antagonists and agonists.

Molecule	Clinical trial #	Target organ	Trial description	Reference
Tolvaptan	NCT01348035	Kidney	Completed; Phase N/A; 30 ADPKD patients. 5-year study. Compare changes in total kidney volume (TKV) with water load.	Clinicaltrials.gov
Tolvaptan	NCT01973140	Kidney	Completed; Phase 4 trial; 17 participants; Determine changes in urine output; hematic AVP and Na^+ (30 or 60 mg tolvaptan).	Clinicaltrials.gov
Tolvaptan	NCT02160145	Kidney	Completed; Phase 3b trial; 1370 ADPKD patients between late stage 2 to early stage 4 CKD, multi-center, randomized. Compare efficacy and safety of tolvaptan (45 to 120 mg/day, split-dose).	Clinicaltrials.gov
Tolvaptan	NCT01638663	Kidney	Completed; Phase 2 trial; 20 healthy participants. Tolvaptan effects on water, Na^+ and K^+ excretion, hematic vasoactive hormones, blood pressure, arterial function with/without NO synthesis inhibition (15 mg/day for 1 day).	Clinicaltrials.gov
Tolvaptan	2006-002768-24	Kidney	Completed; Phase 3 trial; ADPKD patients; multi-center, double-blind. Evaluate tolvaptan long-term efficacy through TKV and hepatotoxicity.	Clinicaltrialsregister.eu
Tolvaptan	NCT01210560	Kidney	Completed; Phase 2 trial; 25 ADPKD patients. Pharmacokinetics and pharmacodynamics, adverse events of immediate vs. modified release formulation (20-120 mg for 7 days).	Clinicaltrials.gov
Tolvaptan	2014-001516-19	Kidney	Completed; Phase 3 trial; ADPKD patients. Evaluate long term safety of titrated immediate-release tolvaptan (OPC 41061, 30-120 mg/day, split dose).	Clinicaltrialsregister.eu
Tolvaptan	NCT00428948	Kidney	Completed; Phase 3 trial; 1445 ADPKD patients. Evaluate 36 months safety and efficacy (45/15 mg, 60/30 mg, or 90/30 mg oral	Clinicaltrials.gov

			tolvaptan for 36 months).	
Tolvaptan	NCT00413777	Kidney	Completed; Phase 2 trial; 46 ADPKD participants. Evaluate 34 months safety, tolerability, efficacy (30-120 mg oral tolvaptan).	Clinicaltrials.gov
Tolvaptan	2010-018401-10	Kidney	Completed; Phase 3 trial; ADPKD patients. Efficacy and safety of long term oral tolvaptan administration.	Clinicaltrialsregister.eu
Tolvaptan	NCT01451827	Kidney	Completed; Phase 2 trial; 178 ADPKD patients; multi-center double-blind. Compare short-term effects of immediate and modified release tolvaptan. (50/80 mg or 60/30 mg daily).	Clinicaltrials.gov
Tolvaptan	NCT02009878	Kidney	Completed; Phase ½, multi-center, double-blind trial; 30 patients with syndrome of inappropriate ADH secretion. Tolvaptan pharmacokinetics, pharmacodynamics and effects on hematic and urine "salt" (3.75-15 mg).	Clinicaltrials.gov
Tolvaptan	2014-000226-38	Kidney	Completed; Phase 3, multi-center, double-blind trial; ADPKD patients with late-stage 2-4 CKD. Tolvaptan efficacy to reduce eGFR decline (45-120 mg/day).	Clinicaltrialsregister.eu
Tolvaptan	NCT03803124	Kidney	Completed; Phase 3 trial; 20 ADPKD patients. Tolvaptan effects on renal blood flow and vasoactive hormones.	Clinicaltrials.gov
Tolvaptan	NCT01336972	Kidney	Completed; Phase 2 trial; 29 ADPKD patients, various levels of renal function. Three-weeks effect on eGFR and TKV of tolvaptan at thehighest tolerated split-dose (45/15 mg-90/30 mg).	Clinicaltrials.gov
Tolvaptan	NCT01280721	Kidney	Completed; Phase 3 trial; multi-center; 135 ADPKD patients enrolled in Trial 156-04-251. Tolvaptan effect on TKV and renal function (Oral administration, 45/15 mg-90/30 mg).	Clinicaltrials.gov

Tolvaptan	NCT01214421	Kidney	Completed; Phase 3 trial, multi-center; 1083 ADPKS patients from trial 156-04-251. Tolvaptan effects on TKV and renal function (45/15 mg-90/30 mg).	Clinicaltrials.gov
Tolvaptan	NCT01430494	Kidney	Completed; Phase N/A; 3409 ADPKD patients. Changes in TKV vs. ADPKD-related outcomes over one year.	Clinicaltrials.gov
Tolvaptan	NCT00841568	Kidney	Completed; Phase 2 trial; 17 ADPKD patients from dose-finding study 156-04-001. Three-year safety and efficacy of tolvaptan/OPC-41061 (15 mg twice daily).	Clinicaltrials.gov
Tolvaptan	NCT01022424	Kidney	Completed; Phase 3 trial; 13 ADPKD patients from study 156-05-002. Administration was continued until tolvaptan/OPC-41061 approval for ADPKD in Japan (15 mg twice daily).	Clinicaltrials.gov
Tolvaptan	GCT03101362	Kidney	Completed; Phase 3; ADPKD patients who enrolled in Trial 156-04-251. Evaluate long-term effects of tolvaptan (45m/15 mg, 60/30mg, 90/30mg, split dose).	Globalclinicaltrialsdata.com
Tolvaptan	NCT03858439	Kidney	Not yet recruiting; Phase N/A; 15 ADPKD patients. Dietary intervention on urine output and quality of life during tolvaptan treatment (15/15-30/30 mg daily).	Clinicaltrials.gov
Tolvaptan	NCT02497521	Kidney	Recruiting; Phase N/A; multi-center; 500 ADPKD patients. Ten-year study of tolvaptan dosage and parameters of ADPKD progression and renal function vs. placebo.	Clinicaltrials.gov
Tolvaptan	NCT02964273	Kidney	Recruiting; Phase 3 trial; multi-center double-blind 1year/open label 2-year; 91 ADPKD children and adolescent patients. Tolvaptan safety, pharmacokinetics, pharmacodynamics in specific age group (15/7.5 mg-45/15 mg, split-dose).	Clinicaltrials.gov
Tolvaptan	NCT02729662	Kidney	In progress; Phase N/A; 118	Clinicaltrials.gov

			ADPKD patients. Five-year tolvaptan efficacy in reducing TKV increase in relation to PKD1, 2 genotype.	
Tolvaptan	NCT03596957	Kidney	Not yet recruiting; Phase 4 trial; 90 ADPKD patients. Measure TKV following tolvaptan (vs. placebo) treatment for six weeks followed by six weeks without medication.	Clinicaltrials.gov
Tolvaptan	NCT02251275	Kidney	Active, not recruiting; Phase 3 trial; multi-center open label trial; 1083 ADPKD patients from 156-13-210, 156-08-271, 156-04-251, or 156-09-290; Establish tolvaptan forty-two weeks safety (15/15 mg-90/30 mg split-dose).	Clinicaltrials.gov
Tolvaptan	2016-000187-42	Kidney	In progress; Phase 3 multi-center double-blind trial; ADPKD patients, children and adolescents. One-year double-blind, plus two-year open label to assess tolvaptan safety, pharmacokinetics, tolerability, efficacy in pediatric patients.	Clinicaltrialsregister.eu
Tolvaptan	2017-004701-40	Kidney	Active, not recruiting; Phase 2 trial; 20 ADPKD patients with normal kidney function. Evaluate the effect of 4-week tolvaptan and octreotide LAR combination therapy vs. tolvaptan and placebo on GFR.	Clinicaltrialsregister.eu
Tolvaptan	NCT02925221	Kidney	Recruiting; Phase N/A; 500 ADPKD patients on tolvaptan therapy. Part of the Health Canada approval requirement for tolvaptan with Patient Reported Outcome Questionnaires, time to renal replacement therapy, long-term mortality.	Clinicaltrials.gov
Tolvaptan	GCT07070402	Kidney	Unknown status; Phase 3 trial; Three-year study of tolvaptan tolerability (45/15- 90/30 mg split-dose).	Globalclinicaltrialsdata.com
Tolvaptan	2017-003864-10	Kidney	Active, not recruiting; Phase 2 pilot study; 12 ADPKD patients; randomized placebo-controlled	Clinicaltrialsregister.eu

			double blind; Assess short-term effects of tolvaptan (120 mg daily).	
Tolvaptan	NCT02847624	Kidney	Recruiting; Phase N/A; 1600 ADPKD patients; evaluate the safety and effectiveness of tolvaptan.	Clinicaltrials.gov
Tolvaptan	NCT03541447	Kidney	Recruiting; Phase 2 trial; 20 ADPKD patients; Evaluate additional short-term effect of Tolvaptan and Octreotide.	Clinicaltrials.gov
Tolvaptan	NCT03949894	Kidney	Active, not recruiting; Phase 4 trial; 100 adult ADPKD Korean patients; Evaluate safety and therapeutic effectiveness of tolvaptan (30mg and 15mg).	Clinicaltrials.gov
Lixivaptan	NCT03487913	Kidney	Active, recruiting; Phase 2 trial; 32 adult ADPKD patients; Evaluate pharmacokinetics and safety of lixivaptan.	Clinicaltrials.gov
Lixivaptan	NCT01056848	Heart	Completed; 135 adult hyponatremia patients; Evaluate safety of lixivaptan.	Clinicaltrials.gov
Lixivaptan	NCT00660959	Heart	Completed; Phase 3 trial; 103 adult hyponatremia patients; Evaluate safety of lixivaptan.	Clinicaltrials.gov
Lixivaptan	NCT01055912	Heart	Completed; Phase 3 trial; 130 HF patients; Evaluate oral lixivaptan in HF patients (100mg once daily).	Clinicaltrials.gov
Lixivaptan	NCT00876798	Heart	Completed; Phase 3 trial; 206 hyponatremia patients; Evaluate safety of oral lixivaptan doses (25, 50 and 100mg daily).	Clinicaltrials.gov
Lixivaptan	NCT00876876	Heart	Terminated; Phase 3 trial; 300 hyponatremia patients; 12-week lixivaptan treatment (25, 50mg).	Clinicaltrials.gov
Lixivaptan	2009-016371-30	Heart	Completed; Phase 3 trial; 150 hyponatremia patients; assess safety of long term lixivaptan treatment; extension of study NCT00578695.	Clinicaltrialsregister.eu
Lixivaptan	NCT00578695	Heart	Completed; Phase 3 trial; 652 adult HF patients; Evaluate safety of lixivaptan.	Clinicaltrials.gov
Lixivaptan	NCT04152837	Kidney	Not recruiting; Phase 3 trial; 50	Clinicaltrials.gov

		Liver	adult ADPKD patients; Evaluate liver function and non-hepatic safety (100-200mg daily).	
Lixivaptan	NCT04064346	Kidney	Not recruiting; Phase 3 trial; 1200 adult ADPKD patients; Evaluate efficacy and safety of lixivaptan (100-200mg twice daily).	Clinicaltrials.gov
Lixivaptan	2008-003191-21	Heart	Completed; Phase 3 trial; 200 adult hyponatremia patients; Evaluate safety and efficacy of lixivaptan.	Clinicaltrialsregister.eu
Lixivaptan	2006-001754-26	Heart	Completed; Phase 3 trial; 100 adult hyponatremia patients; Evaluate safety and efficacy of lixivaptan.	Clinicaltrialsregister.eu
Lixivaptan	NCT03717181	Kidney	Not recruiting; Phase 2 trial; 1 adult ADPKD patient; Evaluate lixivaptan ability to eliminate abdominal pain.	Clinicaltrials.gov
Satavaptan	2006-000134-12	Kidney Liver	Terminated; Phase 3 trial; 250 ADPKD patients; Evaluated efficacy of satavaptan in reducing ascites (5-10 mg daily).	Clinicaltrialsregister.eu
Satavaptan	2006-000135-10	Kidney Liver	Completed; Phase 3 trial; 250 ADPKD patients; Evaluate satavaptan efficacy without concomitant diuretics in reducing ascite recurrence (5-10mg daily).	Clinicaltrialsregister.eu
Satavaptan	NCT00359437	Kidney	Terminated; Phase 3 trial; 501 ADPKD with liver cirrhosis patients; Evaluate efficacy to prevent ascites recurrence (5-10mg daily for 52 weeks).	Clinicaltrials.gov
Satavaptan	2006-000132-27	Liver	Active; Phase 3 trial; 501 patients with liver cirrhosis; Evaluate efficacy of satavaptan on top of medication for liver cirrhosis (5-10mg daily).	Clinicaltrialsregister.eu
Satavaptan	NCT00358878	Liver	Completed; Phase 3 trial; 463 liver cirrhosis and ascites patients; Evaluate efficacy to treat liver cirrhosis and ascites (5-10mg dose daily; 52-week treatment period).	Clinicaltrials.gov
Satavaptan	2006-005753-29	Liver	Active; Phase 2 trial; 80 patients	Clinicaltrialsregist

			with liver cirrhotic ascites; Assess safety and tolerability of continued long-term satavaptan treatment.	er.eu
Conivaptan	NCT01752543	Heart	Completed; Phase 4 trial; 20 HF patients; Evaluated effects of AVP blockade and central hemodynamic system (20 mg bolus followed by infusion of 2 mg/hour).	Clinicaltrials.gov
Conivaptan	NCT00435591	Heart	Completed; Phase 4 trial; 121 hyponatremia patients; Evaluate effectiveness and safety of multiple dosing regimens of IV conivaptan (20 mg/day continuous infusion).	Clinicaltrials.gov
Conivaptan	NCT00843986	Heart	Terminated; Phase 3 trial; 9 patients with acute decompensated heart failure; Evaluate safety and effectiveness of conivaptan (20 mg/day continuous intravenous infusion for 48 hours).	Clinicaltrials.gov
Conivaptan	NCT00478192	Heart	Completed; Phase 3 trial; 50 patients with hypervolemic hyponatremia; Evaluated efficacy and safety of multiple infusions of conivaptan (20 mg twice a day).	Clinicaltrials.gov
Conivaptan	NCT00057356	Heart	Completed; Phase 2 trial; 170 HF patients; randomized, double-blind, placebo-controlled, dose ranging pilot study; Evaluated effects of conivaptan.	Clinicaltrials.gov
Conivaptan	NCT00924014	Kidney	Unknown status; Phase N/A; 8 patients ADPKD; Compare effects of conivaptan and diuretics on renal blood flow and neurohormones (20 mg intravenous).	Clinicaltrials.gov
Conivaptan	NCT00887627	Kidney	Completed; Phase 1 trial; 25 ADPKD patients; Compare conivaptan pharmacokinetics in subjects with mild or moderate	Clinicaltrials.gov

			kidney function (48 hour continuous infusion).	
Conivaptan	NCT00851227	Liver	Completed; Phase 1 trial; 25 patients with liver impairment; Compared 48-hour continuous infusion of conivaptan vs subjects with normal liver function.	Clinicaltrials.gov
Balovaptan	NCT04049578	Brain	Not yet recruiting; Phase 1 trial; 10 patients with Autism Spectrum Disorder (ASD); Evaluate pharmacokinetics, safety and tolerability (Oral dose once a day 6-week treatment period).	Clinicaltrials.gov
Balovaptan	NCT03504917	Brain	Terminated; Phase 3 trial; 350 patients with ASD; Evaluate efficacy, safety, and pharmacokinetics (10 mg of oral administration once a day).	Clinicaltrials.gov
Balovaptan	NCT02901431	Brain	Terminated; Phase 2 trial; 300 ASD patients; Evaluate efficacy and safety (10mg/day for 24 weeks).	Clinicaltrials.gov
Relcovaptan	DOI: 10.1080/09513590400021144	Reproductive system	Completed; 18 women with pre-term pregnancy; Evaluate effect of relcovaptan on uterine contractions in preterm labor (single dose of 400mg relcovaptan).	PubMed
Mozavaptan	DOI: 10.1093/jjco/hyq170	Kidney	Completed; 16 ADH syndrome patients; Evaluated clinical implication of AVPR antagonist mozavaptan hydrochloride (30 mg/day).	PubMed
AVP	NCT01327027	Brain	Completed; Phase 1 trial; 225 healthy participants. AVP effects on emotional and social communication (One of two doses (20 or 40IU)).	Clinicaltrials.gov
AVP	NCT02626832	Brain	Completed; Diagnostic trial (Phase N/A); 25 patients with depression and schizophrenia. NMDA receptor function and AVP effects in these conditions.	Clinicaltrials.gov

AVP	NCT02153502	Brain	Completed; Phase 2 trial; 206 patients suffering from major depressive disorder. Efficacy, safety and tolerability of AVP-786 as an adjunctive therapy for depression (AVP-786 administered twice a day for 10 weeks).	Clinicaltrials.gov
AVP	NCT02394054	Brain	Completed; Early Phase 1 trial; 84 participants; AVP effects on the social brain (i.e. deception detection, empathy, altruism, responses to photo stimuli) (20 IU, intranasal).	Clinicaltrials.gov
AVP	NCT00628550	Heart	Completed; Phase 1 trial; 130 pediatric cardiopulmonary resuscitation patients. Evaluate AVP administration in advanced CPR (One dose of 0.8 units/kg intravenous).	Clinicaltrials.gov
AVP	NCT01247090	Kidney	Completed; Phase 2 trial; 12 participants with chronic hypertension and end stage renal disease (ESRD). AVP effects (0.15 mU/kg/min by infusion at thrice-weekly dialysis treatments for 2 weeks).	Clinicaltrials.gov
Desmopressin	NCT01645475	Brain	Completed; Phase 4 trial; 30 monosymptomatic nocturnal enuresis participants. Impact of desmopressin on ADHD symptoms, cognition, learning, sleep, quality of life and self-esteem.	Clinicaltrials.gov
Desmopressin	NCT02068560	Kidney	Completed; Interventional trial (Phase N/A); 64 healthy participants; Sex differences in the renal sensitivity to desmopressin (Three doses of 0.0003μg/kg, 0.0005μg/kg and 0.004μg/kg, by infusion).	Clinicaltrials.gov
Desmopressin	NCT02937896	Kidney	Completed; Interventional trial (Phase N/A); 40 renal colic patients. Compare efficacy of intranasal desmopressin and intravenous ketorolac (40 μg of desmopressin intranasally and 30	Clinicaltrials.gov

			mg ketorolac intravenous).	
Desmopressin	NCT00748072	Kidney	Completed; Phase 4 trial; 162 participants subject to renal biopsy. Effects of prior desmopressin treatment on post-biopsy bleeding complications (0.3 mcg/kg of desmopressin 1 hour before renal biopsy).	Clinicaltrials.gov
Terlipressin	NCT02059460	Kidney	Completed; Phase 4 trial; 25 participants; Impact of intra and post-operative terlipressin infusion on the occurrence of acute kidney injury (AKI) (1-4 µg/kg/h, continuous infusion).	Clinicaltrials.gov
Terlipressin	NCT03846180	Kidney	Completed; Observational trial (Phase N/A); 1682 cirrhotic patients. Effects of terlipressin on renal function.	Clinicaltrials.gov
Terlipressin	NCT01637454	Kidney Liver	Completed; Phase 3 trial; 46 type 2 hepatorenal syndrome patients. Safety and efficacy of terlipressin and noradrenaline combination therapy (0.5 mg terlipressin every 6 hours, intravenous and continuous infusion of 0.5 mg/h noradrenaline).	Clinicaltrials.gov
Terlipressin	NCT02770716	Kidney Liver	Completed; Phase 3 trial; 300 type 1 hepatorenal syndrome patients. Efficacy and safety (1 mg every 6 hours, intravenous). .	Clinicaltrials.gov
Terlipressin	NCT01932151	Kidney Liver	Completed; Interventional trial (Phase N/A); 18 type 1 hepatorenal syndrome patients. Efficacy and safety (1 mg every 4 hours, intravenous).	Clinicaltrials.gov
Terlipressin	NCT04416282	Kidney Liver	Recruiting; Interventional trial (Phase N/A); 140 hepatorenal syndrome- acute kidney injury patients. Efficacy of terlipressin and albumin combination therapy (2 mg terlipressin every 24 hours, infusion and 1 g/kg/day albumin, intravenous).	Clinicaltrials.gov
Terlipressin	NCT03822091	Kidney Liver	Recruiting; Phase 3 trial; 60 type 1 hepatorenal syndrome patients. Effects of terlipressin infusion alone vs. in combination with	Clinicaltrials.gov

			noradrenaline (2mg every 24 hours, infusion and 0.5 mg/h noradrenaline continuous infusion).	
Felypressin	NCT04236115	N/A	Completed; Phase 4 trial; 95 participants. Felypressin is used as a vasoconstrictor to compare articaine and prilocaine for maxillary teeth extraction (One dose of felypressin of 0.03 IU/ml).	Clinicaltrials.gov
Felypressin	NCT01073371	N/A	Completed; Phase 1 trial; 32 participants. In this study, Felypressin is used as a vasoconstrictor to test the efficacy of prilocaine in maxillary infiltration anesthesia (One dose of felypressin of 0.03 IU/ml).	Clinicaltrials.gov
